# Meal sugar-protein balance determines postprandial FGF21 response in humans

**DOI:** 10.1152/ajpendo.00241.2023

**Published:** 2023-09-20

**Authors:** Stina Ramne, Lisanne Duizer, Mette S. Nielsen, Niklas Rye Jørgensen, Jens S. Svenningsen, Niels Grarup, Anders Sjödin, Anne Raben, Matthew P. Gillum

**Affiliations:** ^1^Novo Nordisk Foundation Center for Basic Metabolic Research, Faculty of Health and Medical Sciences, University of Copenhagen, Copenhagen, Denmark; ^2^Department of Nutrition, Exercise and Sports, Faculty of Science, University of Copenhagen, Frederiksberg, Denmark; ^3^Department of Clinical Biochemistry, Copenhagen University Hospital, Glostrup, Denmark; ^4^Department of Clinical Medicine, University of Copenhagen, Copenhagen, Denmark; ^5^Department of Clinical and Translational Research, Copenhagen University Hospital-Diabetes Center Copenhagen, Herlev, Denmark

**Keywords:** fibroblast growth factor 21, macronutrient balance, meal study, protein, sucrose

## Abstract

Biological mechanisms to promote dietary balance remain unclear. Fibroblast growth factor 21 (FGF21) has been suggested to contribute to such potential regulation considering that FGF21 *1*) is genetically associated with carbohydrate/sugar and protein intake in opposite directions, *2*) is secreted after sugar ingestion and protein restriction, and *3*) pharmacologically reduces sugar and increases protein intake in rodents. To gain insight of the nature of this potential regulation, we aimed to study macronutrient interactions in the secretory regulation of FGF21 in healthy humans. We conducted a randomized, double-blinded, crossover meal study (NCT05061485), wherein healthy volunteers consumed a sucrose drink, a sucrose + protein drink, and a sucrose + fat drink (matched sucrose content), and compared postprandial FGF21 responses between the three macronutrient combinations. Protein suppressed the sucrose-induced FGF21 secretion [incremental area under the curve (iAUC) for sucrose 484 ± 127 vs. sucrose + protein −35 ± 49 pg/mL × h, *P* < 0.001]. The same could not be demonstrated for fat (iAUC 319 ± 102 pg/mL × h, *P* = 203 for sucrose + fat vs. sucrose). We found no indications that regulators of glycemic homeostasis could explain this effect. This indicates that FGF21 responds to disproportionate intake of sucrose relative to protein acutely within a meal, and that protein outweighs sucrose in FGF21 regulation. Together with previous findings, our results suggests that FGF21 might act to promote macronutrient balance and sufficient protein intake.

**NEW & NOTEWORTHY** Here we test the interactions between sugar, protein, and fat in human FGF21 regulation and demonstrate that protein, but not fat, suppresses sugar-induced FGF21 secretion. This indicates that protein outweighs the effects of sugar in the secretory regulation of FGF21, and could suggest that the nutrient-specific appetite-regulatory actions of FGF21 might prioritize ensuring sufficient protein intake over limiting sugar intake.

## Graphical abstract

**Figure:**
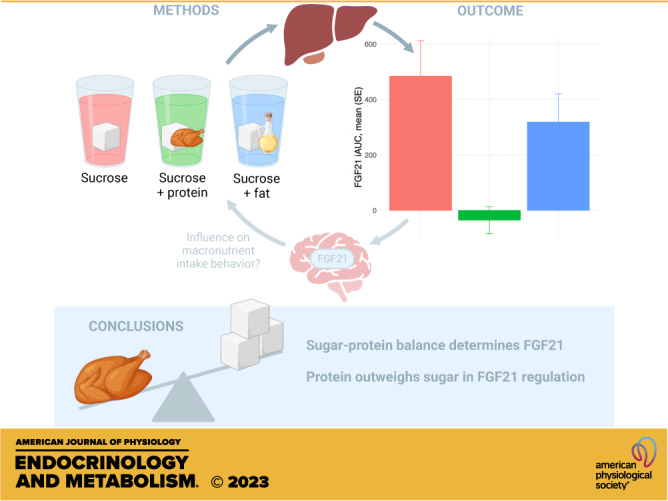

## INTRODUCTION

A balanced dietary intake is a cornerstone in preventing obesity and its cardiometabolic comorbidities. Still, far more is known about the signals that control how much we eat compared with those that determine what we eat. Whether biological mechanisms exist in humans to facilitate a sufficiently balanced intake remains elusive (1, 2). Gaining understanding of such potential mechanisms could have significant implications for human health and disease, as therapies to promote healthier eating habits could become possible by manipulating these pathways.

We hypothesize that the liver-derived hormone fibroblast growth factor 21 (FGF21) could be one of the many possible players involved in enforcing a balanced dietary intake, particularly in terms of macronutrients (3). The most compelling evidence for this comes from genetic association studies, in which genetic variation at the *FGF21* locus is found to be associated with higher carbohydrate and sugar intake and lower protein intake (4–11), as well as with sweet versus savory food liking (12, 13).

Furthermore, FGF21 is hepatically secreted to the circulation after both sugar ingestion and protein restriction. Intake of 75 g of sucrose or fructose leads to an approximate twofold rise in circulating FGF21 2 h after consumption (9, 14–16), whereas intake of glucose results in a smaller FGF21 response (14). Sustained restriction in dietary protein also induces a significant increase in fasting circulating FGF21 levels (17–20), and plasma FGF21 concentrations are reduced postprandially to a high-protein meal (21).

Finally, pharmacological FGF21 administration in rodents reduces sweet taste preference and sugar intake, while encouraging protein intake (22–28). Hence, FGF21 appears to switch intake from sugar to protein via negative feedback signaling to facilitate a balance in macronutrient intake. But there is an ongoing debate whether protein or sugar is the primary determinant or target of FGF21 regulation, and rodent experiments trying to answer this question have reached contradictory conclusions (26–28).

Accordingly, to gain insight of FGF21’s potential role in macronutrient balance regulation, we aimed to study macronutrient interactions in the secretory regulation of FGF21. Using a simple meal study design in healthy human volunteers, we here demonstrate how coingestion of protein or fat with sucrose, in isocaloric amounts, affects the FGF21 secretion, which is known to be induced by ingestion of sucrose. The primary outcome of this study was the FGF21 plasma response after adding protein to a sucrose load, which was compared with the addition of fat as an isocaloric control. The secondary outcomes were postprandial responses in glucose, insulin, glucagon, and subjective appetite ratings following addition of protein or fat to a sucrose load.

## MATERIALS AND METHODS

### Study Design

In this three-arm randomized double-blinded crossover study, participants consumed three different macronutrient combinations on separate days in randomized order. The postprandial FGF21 responses were measured up till 4 h after the test drink. Test days were separated by a washout period of minimum 72 h. The study protocol was approved by the Scientific Ethical Committees of the Capital Region of Denmark (H-21021550) in accordance with the Declaration of Helsinki, and the trial was registered at clinicaltrials.gov (NCT05061485) before study initiation.

### Participants

Healthy men and women within 18–50 yr of age with a body mass index (BMI) of 20–27 kg/m^2^ and self-reported White origin were eligible for the study. Exclusion criteria included any chronic diseases or significant health problems that may interfere with study outcomes as judged by the investigator; use of medication (currently or within the previous 3 mo) that may affect any of the blood parameters assessed in the study as judged by the investigator; blood donation within the last 3 mo; smoking or other nicotine use within the past 3 mo; on a diet or have lost/gained weight within the previous 3 mo (±3 kg); women who are pregnant, breast-feeding, or have the intention of becoming pregnant during the study period; food allergies or food intolerance of the test products; alcohol intake above the recommendations from the Danish Health and Medicines Authority (14 units/week for men and 7 units/week for women); simultaneous participation in other clinical studies that can interfere with the current study; and inability, physically or mentally, to comply with the procedures required by the study protocol. A participant was excluded from the study according to the exclusion criteria or if significant weight changes (±2 kg) occurred between the test days.

Based on a mean increase of 149 ± 161 pg/mL FGF21 from fasting to 120 min after intake of 75 g sucrose (9), we aimed to recruit a study sample of 30 participants to achieve 90% statistical power at a 5% significance level. Participants were recruited via advertisements on webpages for finding study participants between October 2021 and February 2022. Interested participants were contacted and inclusion and exclusion criteria were checked. If eligible, participants were scheduled for an information and screening meeting where informed consent was signed after giving rigorous information about the study. Once consent was signed, height and weight were measured, and the inclusion criterion of a BMI 20–27 kg/m^2^ was checked. Finally, participants were assigned randomly to one of the following blinded test drink sequences: A-B-C, B-C-A, and C-A-B.

### Test Day Procedures

The participants arrived at the study center at the same time on each of the 3 test days, between 8.00 and 9.30 in the morning. At arrival, the participants had fasted for minimum 12 h and been asked to refrain from any alcohol, sweet foods, and strenuous physical exercise the past 24 h. A fasted weight was taken to the nearest 0.1 kg in light clothing, a plastic cannula was inserted in the antecubital vein, and a fasting blood sample was collected. Previously validated subjective appetite ratings based on visual analog scales (VASs) were administered, asking about the desire to eat something sweet, salty, or fatty, as well as perceived satiety, fullness, hunger, thirst, nausea, and prospective food intake (29). After this, the participants drank the test drink within 10 min but not faster than 5 min, and aimed at consuming the drinks equally fast on all 3 test days. After the drink was finished, VASs were filled out after 30, 60, 120, 180, and 240 min and blood samples were taken after 60, 120, 180, and 240 min. After the last blood sample, the cannula was removed, and the participants were given a small snack before they were sent home.

### Test Drinks

The three test drinks all contained 75 g of sucrose diluted in 250 mL water. The sucrose + protein drink had 25 g of whey protein added, corresponding to 100 kcal protein. The sucrose + fat drink had 28 g of heavy cream (38% fat), corresponding to 11 g of fat and 100 kcal. Hence, all three drinks were matched for sucrose content, and the test drinks with added protein or fat were matched for energy content. All drinks also contained a sugar-free cordial mix for flavor. The drinks were served in nontransparent bottles, so it was not visible which drink was served to either researchers or participants. The drinks were further blinded with the labels A, B, and C throughout the study and were not revealed until after data analysis.

### Blood Sample Analyses

Plasma and serum samples were stored at −80°C and thawed on wet ice on the day of analysis. Intact FGF21 was measured in duplicate in EDTA plasma samples by enzyme-linked immunosorbent assay (ELISA) (Eagle Biosciences, F2131-K01) in accordance with the manufacturer’s instructions. All samples from the same individual were analyzed on the same 96-well plate to avoid any potential within-individual batch effects. FGF21 concentrations below the lowest standard 32 pg/mL (16% of samples) were set to 0.5 × 32.5 = 16.25 pg/mL. FGF21 concentrations above the highest standard of 2,000 pg/mL (10% of all samples) were very consistent within individuals and thus considered valid measurements and used in further analyses.

Glucose was measured in fluoride plasma samples on the VITROS 4600/5600 automatic analyzer (Ortho Clinical Diagnostics, Raritan, NJ). All samples were measured in the same batch. Intermediary precision expressed as a coefficient of variation was determined using the manufacturer’s control specimens and was below 2.5% at glucose levels of both 5.0 and 16.0 mmol/L.

Serum insulin was determined using the Liaison Insulin assay on the Liaison XL automatic analyzer (DiaSorin, Saluggia, Italy). The assay is a chemiluminescent immunoassay. All samples were measured in one batch. Intermediary precision was below 8% as stated in the manufacturer’s instructions for use.

Glucagon was measured in EDTA plasma samples by ELISA (Mercodia, 10-1271-01) in accordance with the manufacturer’s instructions. Plasma glucagon concentrations below the limit of quantification (1.48 pmol/L) were substituted with 0.5 × 1.48 pmol/L.

Triglycerides (Sigma-Aldrich, MAK266), cholesterol (Sigma-Aldrich, MAK043), and γ-glutamyl transferase (GGT) (Sigma-Aldrich, MAK089) were all measured in fasting serum samples from the participants first test day by colorimetric assay kits in accordance with the manufacturer’s instructions. For GGT, all samples were below the limit of quantification (with the lowest standard of 8 nmol/well and a 33-min incubation at 37°C) using undiluted serum. All absorbances for ELISA and colorimetric assays were measured using a Hidex Sense microplate reader.

### Statistical Analysis

Linear mixed models (LMMs) exploring a test drink × time interaction with study individuals treated as random effects and adjustment for age and sex were performed for the repeated measurements of FGF21, glucose, insulin, glucagon, and all nine subjective appetite ratings. Pairwise comparisons were further conducted on these LMMs if significant drink × time interactions were found. Both absolute concentrations and fold changes were examined log-transformed for FGF21, glucose, insulin, and glucagon. However, one FGF21 fold change observation was excluded from analysis as it constituted an extreme outlier (the concentrations in itself were not unreasonable), unless when the FGF21 fold changes were log-transformed. iAUCs were calculated using the trapezoidal method for all studied outcomes. The iAUCs were compared between test drinks using LMM, treating individuals as random effects and adjusting for age and sex. Association between iAUCs of FGF21 response and iAUCs of subjective appetite ratings was investigated using linear regressions adjusted for age and sex.

## RESULTS

**Figure 1.**
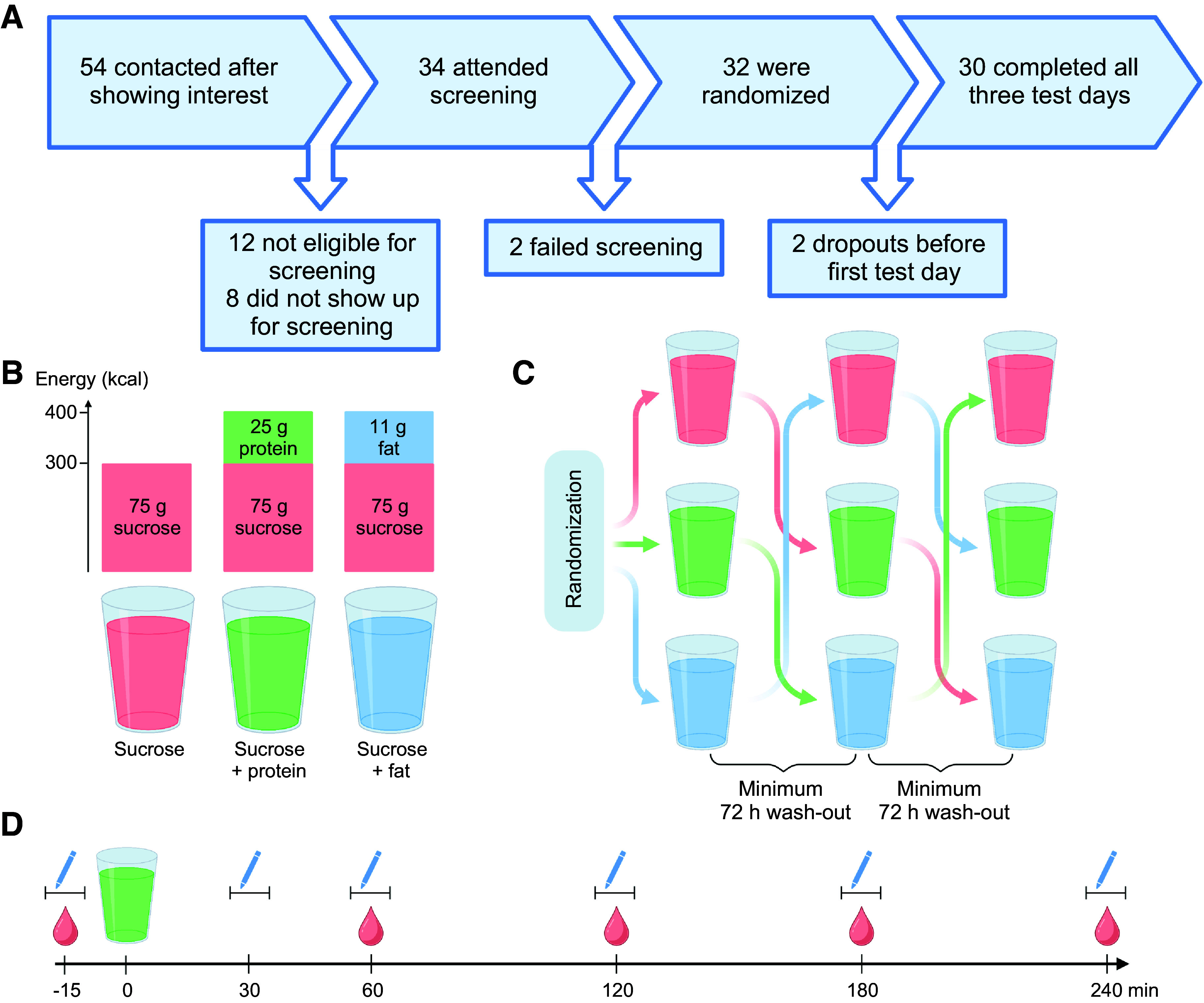
Overview of the study design. *A*: flowchart of the process that resulted in the study sample. *B*: description of the three test drinks. *C*: overview of the crossover study design. *D*: timeline of the test day procedure. Created with BioRender.com.

Out of the 54 individuals who were contacted after showing interest in participating in the meal study, 34 individuals attended the screening visit. Of these, 32 were eligible and consequently randomized to a test drink sequence after signing an informed consent form. Two individuals dropped out before the first test day, leaving 30 individuals who participated in the study, all of whom completed all 3 test days (Fig. 1). Study participants were 57% women, young (means ± SD, age 26.4 ± 6.3 yr), lean (BMI 22.9 ± 2.0 kg/m^2^), and had glycemic and lipid markers within normal ranges (Table 1).

**Table 1.** Participant characteristics of the study sample

Participant Characteristics	Means (SD)*
*n*	30
Sex, women	57%
Age, yr	26.4 (6.3)
BMI, kg/m^2^	22.9 (2.0)
Fasting FGF21, pg/mL	160 (90–434)^1^
Fasting glucose, mmol/L	5.3 (0.2)
Fasting insulin, pmol/L	42.8 (17.9)
Fasting glucagon, pmol/L	7.0 (3.8)
Fasting cholesterol, mmol/L	3.8 (0.2)
Fasting triglycerides, mmol/L	0.19 (0.07)
Fasting GGT, mU/mL	All below LoQ

Baseline characteristics of the study sample measured at the first test day. BMI, body mass index; GGT, γ-glutamyl transferase; IQR, interquartile range, LoQ, limit of quantification.

*Data are presented as means (SD), unless stated otherwise;

^1^Median (IQR).

Fasting FGF21 concentrations were highly varying in the study sample, with five individuals showing high FGF21 concentrations above 1,000 pg/mL (consistently on all 3 test days). As a result of this high variation, the median and interquartile range (IQR) of fasting FGF21 concentrations across all 3 test days were 177 (77–459) pg/mL with a mean ± SD of 495 ± 73 pg/mL. None of the assessed baseline characteristics could explain this variation in fasting FGF21 concentrations.

### Protein Suppresses the Sucrose-Induced FGF21 Response

**Figure 2.**
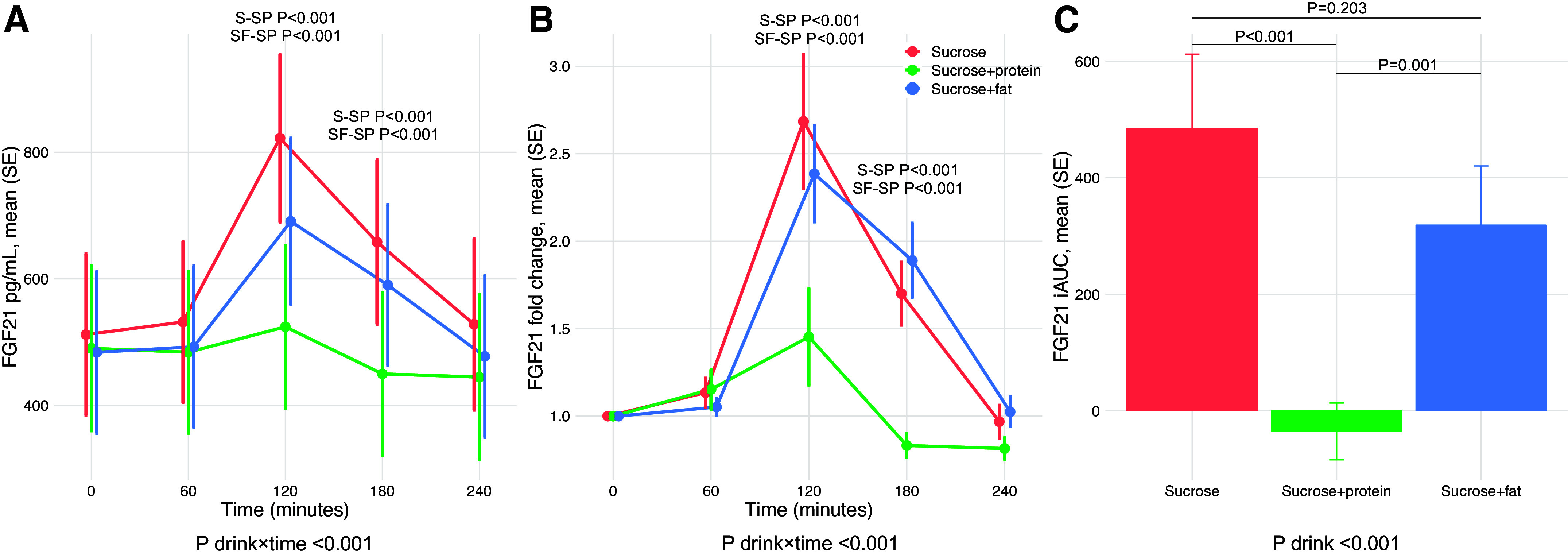
Postprandial plasma FGF21 response up to 4 h after the three test drinks (primary outcome). For each of the three test drinks [sucrose (S), sucrose + protein (SP), and sucrose + fat (SF)], *A* displays the means (SE) of absolute concentrations in FGF21 across the five time points, *B* displays the means (SE) of fold changes in FGF21 concentrations from fasting at time point 0, and *C* displays the means (SE) of FGF21 iAUCs. Graphs show crude means (SE) values, while all *P* values are determined using LMM adjusted for age and sex with individuals treated as random effects. Repeated measurements analyses are performed with log-transformed FGF21 values for both absolute concentrations and fold changes. In *A* and *B*, only significant *P* values for pairwise comparisons are presented. FGF21, fibroblast growth factor 21; iAUC, incremental area under the curve; LMM, linear mixed model.

Our primary outcome was to compare the postprandial FGF21 responses between the three macronutrient combinations. As shown in Fig. 2, *A* and *B* and Supplemental Table S1, significant drink × time interactions were observed for both the absolute FGF21 concentrations (*P* < 0.001) and fold change from fasting in FGF21 concentration (*P* < 0.001). Both absolute concentrations and fold changes in FGF21 were lower after the sucrose + protein drink compared with both the sucrose drink and the sucrose + fat drink at both 120 and 180 min (*P* < 0.001). After the sucrose + protein drink only, the average FGF21 concentrations descended below baseline already after 180 min. The average FGF21 response expressed as incremental areas under the curve (iAUCs) was smaller after the sucrose + protein drink (means ± SD −35 ± 49 pg/mL × h, *P* < 0.001) compared with the sucrose drink (484 ± 127 pg/mL × h) and the sucrose + fat drink (319 ± 102 pg/mL × h, *P* = 0.001). There was no significant difference in the FGF21 iAUCs between the sucrose drink and the sucrose + fat drink (*P* = 0.203) (Fig. 2*C* and Supplemental Table S2). We further made the observation that the overall FGF21 responses to the test drinks were much reduced among the individuals with very high fasting FGF21 concentrations (Supplemental Fig. S1). All in all, these results show that protein suppresses the FGF21 response induced by sucrose, whereas the same is not the case for fat.

### The Suppressing Effect of Protein on FGF21 Does Not Appear to Be Attributed to Glycemic Regulation

We next assessed the postprandial glucose, insulin, and glucagon responses to the three test drinks to evaluate whether the suppressing effects of protein on sucrose-induced FGF21 secretion are rather a consequence of the nutrients themselves or explained by alterations in the metabolic fate of the different macronutrient combinations.

**Figure 3.**
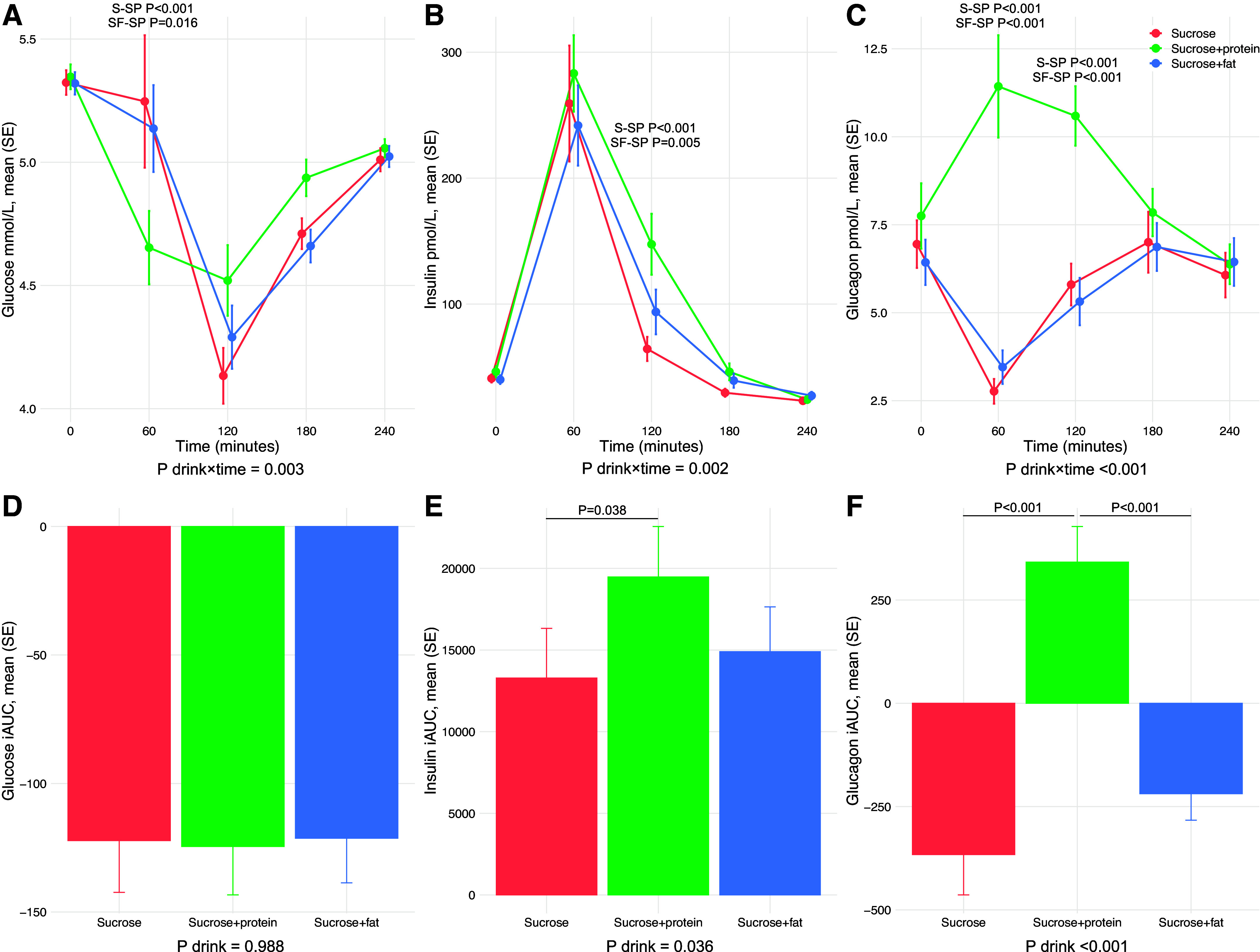
Postprandial glucose, insulin, and glucagon responses up to 4 hours after the three test drinks (secondary outcomes). For each of the three test drinks [sucrose (S), sucrose + protein (SP), and sucrose + fat (SF)], *A*–*C* displays the mean (SE) of absolute concentrations across the five time points: glucose (*A*), insulin (*B*), and glucagon (*C*). The means (SE) of the iAUCs calculated from the postprandial responses: glucose (*D*), insulin (*E*), and glucagon (*F*). Graphs show crude means (SE) values, while all *P* values are determined using LMM adjusted for age and sex with individuals treated as random effects. Analyses of repeated measurements are performed with log-transformed values of glycemic markers. Only significant *P* values for pairwise comparisons are presented.

Although we observed a significant drink × time interaction on glucose concentrations (*P* = 0.003), with lower glucose concentrations observed after the sucrose + protein drink compared with both the sucrose drink (*P* < 0.001) and the sucrose + fat (*P* = 0.016) drink at 60 min (Fig. 3*A* and Supplemental Table S1), there was no difference in glucose iAUC between the test drinks (*P* = 0.988) (Fig. 3*D* and Supplemental Table S2). We also found a significant drink × time interaction on insulin concentrations (*P* = 0.002), with slightly higher insulin concentrations observed after the sucrose + protein drink compared with both the sucrose drink and the sucrose + fat drink at 120 min (Fig. 3*B* and Supplemental Table S1). Insulin iAUCs were also different between the test drinks (*P* = 0.036), showing somewhat higher insulin iAUC after the sucrose + protein drink compared with the sucrose drink in pairwise comparisons (*P* = 0.038) (Fig. 3*E* and Supplemental Table S2). However, the assessments of glucose and insulin are hindered by the fact that the first postprandial measurements were taken only after 60 min.

Although glucagon concentrations were reduced after both the sucrose drink and the sucrose + fat drink, a positive glucagon response curve was observed after the sucrose + protein drink. As such, there was a significant drink × time interaction on glucagon concentrations (*P* < 0.001), with higher glucagon concentrations observed after the sucrose + protein drink compared with both the sucrose drink and the sucrose + fat drink at both 60 and 120 min (Fig. 3*C* and Supplemental Table S1). These differences were also reflected by higher glucagon iAUCs after the sucrose + protein drink (*P* < 0.001), whereas there were no differences between the sucrose drink and the sucrose + fat drink (Fig. 3*F* and Supplemental Table S2). This means that the condition generating the highest glucagon response (the sucrose + protein drink) also generated a suppressed FGF21 response, contradicting previous observations of glucagon inducing FGF21 secretion (30–34).

### Effect of Postprandial FGF21 Response on Appetite and Food Wanting Remains Elusive

We further assessed nine different postprandial appetite and food wanting sensations using VAS after consumption of the three test drinks. A significant drink × time interaction was observed for fullness (*P* = 0.007) (Supplemental Fig. S2 and Supplemental Table S3), which was reported higher after the sucrose + protein drink compared with the sucrose drink at 30 and 60 min in pairwise comparisons (*P* = 0.012 and *P* = 0.011, respectively). Based on the calculated iAUCs of these subjective appetite ratings, a significant effect of the test drink was observed for desire to eat something fatty (*P* = 0.017) (Supplemental Fig. S2 and Supplemental Table S3), demonstrating lower desire after the sucrose + protein drink compared with the sucrose drink in pairwise comparisons (*P* = 0.020). We could also demonstrate a significant association between iAUCs of FGF21 concentrations and iAUCs of reported desire to eat something fatty (corresponding to a higher desire for fat with higher FGF21 response, β = −0.15, *P* = 0.038) (Supplemental Fig. S2). No other appetite ratings were different between the test drinks or associated the FGF21 iAUCs.

## DISCUSSION

Using a straightforward meal study design, we have shown that a simultaneous intake of protein suppresses the FGF21 response triggered by sucrose in healthy humans. This suppressing effect was specific for protein and was not observed when an isocaloric amount of fat was consumed in combination with sucrose. These results provide new insights into how we understand the effect of macronutrient composition on the secretory regulation of FGF21 in humans: FGF21 responds to sucrose-protein balance rather than the single nutritional stimuli, and protein intake outweighs sucrose intake in the secretory regulation of FGF21.

### FGF21 Responds to Sucrose-Protein Balance Acutely within a Meal

We here demonstrate a sugar and protein interaction in which FGF21 secretion is induced by disproportionate intake of sugar relative to protein. The fact that protein has an acute inhibiting effect on FGF21 secretion (21), in addition to that maintained protein restriction induces FGF21 secretion (17–20), provides new insights to previous hypotheses regarding FGF21 secretory regulation. That a macronutrient imbalance is sensed and reacted to acutely, even before it has become a negative nitrogen imbalance and/or a hepatic stress, suggests a mechanism that is motivated by maintaining macronutrient intake homeostasis [similar to the “wisdom of the body” hypothesis (1, 2)] rather than correcting such conditions.

### Protein Outweighs Sugar in Determining FGF21 Secretion

About 75 g of sucrose, a dose that is 50% higher than the maximum recommended intake of added sugar over an entire day (10E% on a 2,000-kcal diet) (35, 36), was trivial for FGF21 secretion when protein was present, even at a protein dose of one-third of the sucrose dose (25 g vs. 75 g). However, the mechanism behind how protein can overrule the sugar-induced signaling pathway that activates FGF21 expression and secretion is not yet understood.

Previous research has pointed to general control nonderepressible 2 (GCN2) as a sensor of amino acid deficiency that, via eukaryotic initiation factor 2α (eIF2α) phosphorylation and activating transcription factor 4 (ATF4), induces FGF21 secretion following protein restriction as part of the integrated stress response (ISR) (17, 37, 38). Nuclear protein 1 (NUPR1) has also been identified as a mediator of FGF21 induction in depletion of nonessential amino acids specifically (18). It is intuitive to speculate that the same mechanisms may also sense the presence of protein and act to downregulate FGF21 after protein intake. However, other major amino acid sensors, such as the mechanistic target of rapamycin complex 1 (mTORC1) (39, 40), warrant further research in relation to FGF21 regulation. Furthermore, whether these pathways can interact with the carbohydrate-responsive element-binding protein (ChREBP), which is responsible for the FGF21 induction by carbohydrates (22, 41), remains uncertain. However, the ChREBP pathway has been demonstrated to also require peroxisome proliferator-activated receptor α (PPARα) (42), and PPARα also appears to partly mediate the FGF21 response to protein-restricted diets (17). Further considering that mTORC1 activation has been shown to inhibit PPARα (43), and mTORC1 is largely activated by amino acids (39), this could make up a potential mechanism of how the presence of protein could suppress sugar-induced FGF21 secretion. Such a potential pathway does, however, remain unstudied, and disentangling this will be an important research question for future mechanistic studies.

There also remains a possibility that the varying FGF21 responses to the test drinks in this study are an effect by altered clearance of FGF21 [renally or by fibroblast activation protein (44)] rather than by altered secretion. Although this alternative appears less likely, given the known transcription factor activation of FGF21 by nutrient exposures mentioned earlier.

### Is the Priority of FGF21 to Ensure Sufficient Protein Intake?

Genetic support for FGF21 having a meaningful impact on dietary preferences and intake in humans is provided by genome-wide association studies, which consistently identify *FGF21* variants to be associated with higher carbohydrate and sugar intake but lower protein intake (4–8), as well as with sweet versus savory food liking (12, 13). As our results indicate that protein is a stronger determinant of FGF21 secretion over sugar/carbohydrates, this suggests that FGF21’s primary effect on preferences and intake may rather be to ensure sufficient protein intake over limiting sugar intake. Such a prioritization would fit well with the protein leverage hypothesis (45). Still, mice studies have reached contradictory conclusions when attempting to answer whether FGF21’s main action is primarily directed toward regulating intake of protein versus carbohydrates (26–28). Nevertheless, there exists no convincing reason for why the effects of FGF21 cannot be dually directed to target preferences of both these nutrients, to achieve a balanced intake between the two.

Considering these potential feedback effects of FGF21 on dietary preferences, we could in this study demonstrate that the FGF21 responses from the different drinks resulted in an effect on VAS-measured postprandial desire for fatty foods (which may coincide with protein-rich savory foods), but not for sweet foods. This observed effect could, however, potentially be explained by the fact that the sucrose + protein drink also yielded higher fullness, which is what can be expected from a protein addition of 25 g (46).

### Glucagon Does Not Explain Protein’s Suppressing Effect on Sucrose-Induced FGF21

We additionally demonstrate that the glucagon response was in the opposite direction following the sucrose-protein drink compared with the sucrose and sucrose + fat drink. This positive glucagon curve is not unexpected, as it is well-known that amino acids stimulate glucagon secretion (47, 48), even to a degree that protein can overrule the insulin-stimulated downregulation of glucagon after a carbohydrate meal (49). This is interesting considering that previous studies have indicated that glucagon can stimulate FGF21 secretion through a wide range of different mechanisms (30–34). Contrastingly, in our study, the sucrose + protein drink generated both an elevation in glucagon concentrations and a suppressed FGF21 response, indicating that glucagon was most likely not involved in generating the FGF21 responses observed in this study.

### Strengths and Limitations

Many previous studies in both rodents and humans attempting to study the relation between macronutrients and FGF21 have been limited by isocaloric macronutrient substitutions to draw firm conclusions (17–20), as it also has been important to distinguish the effects of altered macronutrients from the effects of altered energy intake. A major strength in our study is that we have not substituted sucrose for protein, showing that protein has an independent suppressing effect on FGF21 secretion. We have at the same time controlled for energy by comparing with an isocaloric amount of fat, showing that the suppressing effect of protein was independent of energy.

A limitation of the current investigation is, however, that it has only been performed using whey protein. Given that whey is a particularly rapidly metabolized and insulinemic protein (50, 51), it is uncertain whether our results are generalizable to other amino acid compositions. In addition, the fact that the first postprandial measurements were taken first after 60 min limits our interpretation of the glucose and insulin curves.

Finally, regarding generalizability of the findings from this exclusively lean study sample, since postprandial FGF21 responses to sugars have been shown to be elevated in individuals with obesity (14), but human FGF21 responses to low protein diets have not been specifically studied in obesity, it remains uncertain how sugar and protein would interact in the setting of obesity. A case for FGF21 resistance in obesity has been debated (52), but how that potentially would influence FGF21 responses to nutritional stimuli, or FGF21’s influence on macronutrient preferences, requires future investigation.

### Conclusions

This human study demonstrates an acute role of macronutrient intake imbalance in the secretory regulation of FGF21 as coingestion of protein suppressed the postprandial FGF21 secretion induced by sucrose. This further illustrates that protein outweighs the effect of sucrose on FGF21 secretion. Considering previous findings of FGF21’s impact on diet preferences in mice, and the associations between *FGF21* genetic variants and macronutrient intake, this suggests a mechanism of autoregulation of macronutrient intake to promote diet balance, perhaps focused on avoiding insufficient protein intake. Given that many interspecies differences in FGF21 metabolism have been identified, it is of great importance to demonstrate a priority for protein over sugar in FGF21 regulation in humans, as has been hypothesized from previous mouse studies (53). However, future studies are necessary to understand the details of the sensing mechanisms, how protein can outweigh the sugar-induced signaling pathways stimulating FGF21 secretion, and how FGF21 may correct an imbalanced intake by shifting macronutrient preference and intake in humans.

## DATA AVAILABILITY

Data will be made available upon reasonable request.

## SUPPLEMENTAL DATA

10.6084/m9.figshare.24057426.v1 Supplemental Tables S1–S3 and Supplemental Figs. S1 and S2: https://doi.org/10.6084/m9.figshare.24057426.v1.

## GRANTS

This study was supported by a grant from the Independent Research Fund Denmark (Grant Number 0134-00375B). The Novo Nordisk Foundation Center for Basic Metabolic Research is funded by an unconditional donation from the Novo Nordisk Foundation (Grant Number NNF18CC0034900).

## DISCLOSURES

A.R. has received speaker’s honoraria from Nestlé, Unilever A/S, and the International Sweeteners Association. M.P.G. and M.S.N. initiated employments at Novo Nordisk A/S during the process of this work. None of the other authors has any conflicts of interest, financial or otherwise, to disclose.

## AUTHOR CONTRIBUTIONS

S.R., M.S.N., A.S., A.R., and M.P.G. conceived and designed research; S.R., L.D., and M.S.N. performed experiments; S.R., L.D., N.R.J., and J.S.S. analyzed data; S.R., L.D., M.S.N., N.R.J., J.S.S., N.G., A.S., A.R., and M.P.G. interpreted results of experiments; S.R. prepared figures; S.R. and L.D. drafted manuscript; S.R., L.D., M.S.N., N.R.J., J.S.S., N.G., A.S., A.R., and M.P.G. edited and revised manuscript; S.R., L.D., M.S.N., N.R.J., J.S.S., N.G., A.S., A.R., and M.P.G. approved final version of manuscript.

## References

[1] Berthoud H-R, Seeley RJ (Editors). Neural and Metabolic Control of Macronutrient Intake. Boca Raton, FL: CRC Press, 1999.

[2] Berthoud HR, Münzberg H, Richards BK, Morrison CD. Neural and metabolic regulation of macronutrient intake and selection. Proc Nutr Soc 71: 390–400, 2012. doi:10.1017/S0029665112000559. 22617310 PMC3617924

[3] Flippo KH, Potthoff MJ. Metabolic messengers: FGF21. Nat Metab 3: 309–317, 2021. doi:10.1038/s42255-021-00354-2. 33758421 PMC8620721

[4] Chu AY, Workalemahu T, Paynter NP, Rose LM, Giulianini F, Tanaka T, Ngwa JS; CHARGE Nutrition Working Group; Qi Q, Curhan GC, Rimm EB, Hunter DJ, Pasquale LR, Ridker PM, Hu FB, Chasman DI, Qi L; DietGen Consortium. Novel locus including FGF21 is associated with dietary macronutrient intake. Hum Mol Genet 22: 1895–1902, 2013. doi:10.1093/hmg/ddt032. 23372041 PMC3612009

[5] Tanaka T, Ngwa JS, van Rooij FJ, Zillikens MC, Wojczynski MK, Frazier-Wood AC, et al. Genome-wide meta-analysis of observational studies shows common genetic variants associated with macronutrient intake. Am J Clin Nutr 97: 1395–1402, 2013. doi:10.3945/ajcn.112.052183. 23636237 PMC3652928

[6] Merino J, Dashti HS, Li SX, Sarnowski C, Justice AE, Graff M, et al. Genome-wide meta-analysis of macronutrient intake of 91,114 European ancestry participants from the cohorts for heart and aging research in genomic epidemiology consortium. Mol Psychiatry 24: 1920–1932, 2019. doi:10.1038/s41380-018-0079-4. 29988085 PMC6326896

[7] Meddens SFW, de Vlaming R, Bowers P, Burik CAP, Linner RK, Lee C, et al. Genomic analysis of diet composition finds novel loci and associations with health and lifestyle. Mol Psychiatry 26: 2056–2069, 2021. doi:10.1038/s41380-020-0697-5. 32393786 PMC7767645

[8] Merino J, Dashti HS, Sarnowski C, Lane JM, Todorov PV, Udler MS, Song Y, Wang H, Kim J, Tucker C, Campbell J, Tanaka T, Chu AY, Tsai L, Pers TH, Chasman DI, Rutter MK, Dupuis J, Florez JC, Saxena R. Genetic analysis of dietary intake identifies new loci and functional links with metabolic traits. Nat Hum Behav 6: 155–163, 2022. doi:10.1038/s41562-021-01182-w. 34426670 PMC8799527

[9] Søberg S, Sandholt CH, Jespersen NZ, Toft U, Madsen AL, von Holstein-Rathlou S, Grevengoed TJ, Christensen KB, Bredie WLP, Potthoff MJ, Solomon TPJ, Scheele C, Linneberg A, Jørgensen T, Pedersen O, Hansen T, Gillum MP, Grarup N. FGF21 is a sugar-induced hormone associated with sweet intake and preference in humans. Cell Metab 25: 1045–1053.e6, 2017. doi:10.1016/j.cmet.2017.04.009. 28467924

[10] Janzi S, González-Padilla E, Najafi K, Ramne S, Ahlqvist E, Borné Y, Sonestedt E. Single nucleotide polymorphisms in close proximity to the fibroblast growth factor 21 (FGF21) gene found to be associated with sugar intake in a Swedish population. Nutrients 13: 3954, 2021. doi:10.3390/nu13113954. 34836209 PMC8622171

[11] Frayling TM, Beaumont RN, Jones SE, Yaghootkar H, Tuke MA, Ruth KS, Casanova F, West B, Locke J, Sharp S, Ji Y, Thompson W, Harrison J, Etheridge AS, Gallins PJ, Jima D, Wright F, Zhou Y, Innocenti F, Lindgren CM, Grarup N, Murray A, Freathy RM, Weedon MN, Tyrrell J, Wood AR. A common allele in FGF21 associated with sugar intake is associated with body shape, lower total body-fat percentage, and higher blood pressure. Cell Rep 23: 327–336, 2018. doi:10.1016/j.celrep.2018.03.070. 29641994 PMC5912948

[12] May-Wilson S, Matoba N, Wade KH, Hottenga JJ, Concas MP, Mangino M, Grzeszkowiak EJ, Menni C, Gasparini P, Timpson NJ, Veldhuizen MG, de Geus E, Wilson JF, Pirastu N. Large-scale GWAS of food liking reveals genetic determinants and genetic correlations with distinct neurophysiological traits. Nat Commun 13: 2743, 2022. doi:10.1038/s41467-022-30187-w. 35585065 PMC9117208

[13] Fayzullina S, Smith RP, Furlotte N, Hu Y, Hinds D, Tung JY. White Paper 23 ‐ 08: Genetic Associations with Traits in 23andMe Customers. 2015. permalinks.23andme.com/pdf/23-08_genetic_associations_with_traits.pdf [2023 Mar 30].

[14] Dushay JR, Toschi E, Mitten EK, Fisher FM, Herman MA, Maratos-Flier E. Fructose ingestion acutely stimulates circulating FGF21 levels in humans. Mol Metab 4: 51–57, 2015. doi:10.1016/j.molmet.2014.09.008. 25685689 PMC4314524

[15] Migdal A, Comte S, Rodgers M, Heineman B, Maratos-Flier E, Herman M, Dushay J. Fibroblast growth factor 21 and fructose dynamics in humans. Obes Sci Pract 4: 483–489, 2018 [Erratum in *Obes Sci Pract* 5: 91, 2019]. doi:10.1002/osp4.295. 30338119 PMC6180711

[16] Alves JM, Yunker AG, Luo S, Jann K, Angelo B, DeFendis A, Pickering TA, Smith A, Monterosso JR, Page KA. FGF21 response to sucrose is associated with BMI and dorsal striatal signaling in humans. Obesity (Silver Spring) 30: 1239–1247, 2022. doi:10.1002/oby.23432. 35491674 PMC11798647

[17] Laeger T, Henagan TM, Albarado DC, Redman LM, Bray GA, Noland RC, Münzberg H, Hutson SM, Gettys TW, Schwartz MW, Morrison CD. FGF21 is an endocrine signal of protein restriction. J Clin Invest 124: 3913–3922, 2014. doi:10.1172/JCI74915. 25133427 PMC4153701

[18] Maida A, Zota A, Sjøberg KA, Schumacher J, Sijmonsma TP, Pfenninger A, Christensen MM, Gantert T, Fuhrmeister J, Rothermel U, Schmoll D, Heikenwälder M, Iovanna JL, Stemmer K, Kiens B, Herzig S, Rose AJ. A liver stress-endocrine nexus promotes metabolic integrity during dietary protein dilution. J Clin Invest 126: 3263–3278, 2016. doi:10.1172/JCI85946. 27548521 PMC5004939

[19] Gosby AK, Lau NS, Tam CS, Iglesias MA, Morrison CD, Caterson ID, Brand-Miller J, Conigrave AD, Raubenheimer D, Simpson SJ. Raised FGF-21 and triglycerides accompany increased energy intake driven by protein leverage in lean, healthy individuals: a randomised trial. PLoS One 11: e0161003, 2016. doi:10.1371/journal.pone.0161003. 27536869 PMC4990330

[20] Vinales KL, Begaye B, Bogardus C, Walter M, Krakoff J, Piaggi P. FGF21 is a hormonal mediator of the human “thrifty” metabolic phenotype. Diabetes 68: 318–323, 2019. doi:10.2337/db18-0696. 30257977 PMC6341300

[21] Herpich C, Haß U, Kochlik B, Franz K, Laeger T, Klaus S, Bosy-Westphal A, Norman K. Postprandial dynamics and response of fibroblast growth factor 21 in older adults. Clin Nutr 40: 3765–3771, 2021. doi:10.1016/j.clnu.2021.04.037. 34130022

[22] von Holstein-Rathlou S, BonDurant LD, Peltekian L, Naber MC, Yin TC, Claflin KE, Urizar AI, Madsen AN, Ratner C, Holst B, Karstoft K, Vandenbeuch A, Anderson CB, Cassell MD, Thompson AP, Solomon TP, Rahmouni K, Kinnamon SC, Pieper AA, Gillum MP, Potthoff MJ. FGF21 mediates endocrine control of simple sugar intake and sweet taste preference by the liver. Cell Metab 23: 335–343, 2016. doi:10.1016/j.cmet.2015.12.003. 26724858 PMC4756759

[23] Talukdar S, Owen BM, Song P, Hernandez G, Zhang Y, Zhou Y, Scott WT, Paratala B, Turner T, Smith A, Bernardo B, Müller CP, Tang H, Mangelsdorf DJ, Goodwin B, Kliewer SA. FGF21 regulates sweet and alcohol preference. Cell Metab 23: 344–349, 2016. doi:10.1016/j.cmet.2015.12.008. 26724861 PMC4749404

[24] Jensen-Cody SO, Flippo KH, Claflin KE, Yavuz Y, Sapouckey SA, Walters GC, Usachev YM, Atasoy D, Gillum MP, Potthoff MJ. FGF21 signals to glutamatergic neurons in the ventromedial hypothalamus to suppress carbohydrate intake. Cell Metab 32: 273–286.e6, 2020. doi:10.1016/j.cmet.2020.06.008. 32640184 PMC7734879

[25] Stevanovic DM, Hebert AJ, Desai BN, Singhal G, Adams AC, Flier JS, Maratos-Flier E. Fibroblast growth factor 21 (FGF21) creates sugar-specific taste aversion to fructose through action in the brain in mice (Preprint). bioRxiv, 2020. doi:10.1101/2020.01.27.921361.

[26] Flippo KH, Jensen-Cody SO, Claflin KE, Potthoff MJ. FGF21 signaling in glutamatergic neurons is required for weight loss associated with dietary protein dilution. Sci Rep 10: 19521, 2020. doi:10.1038/s41598-020-76593-2. 33177640 PMC7658965

[27] Larson KR, Chaffin AT, Goodson ML, Fang Y, Ryan KK. Fibroblast growth factor-21 controls dietary protein intake in male mice. Endocrinology 160: 1069–1080, 2019. doi:10.1210/en.2018-01056. 30802283 PMC6469953

[28] Wu CT, Larson KR, Goodson ML, Ryan KK. Fibroblast growth factor 21 and dietary macronutrient intake in female mice. Physiol Behav 257: 113995, 2022. doi:10.1016/j.physbeh.2022.113995. 36240865

[29] Flint A, Raben A, Blundell JE, Astrup A. Reproducibility, power and validity of visual analogue scales in assessment of appetite sensations in single test meal studies. Int J Obes Relat Metab Disord 24: 38–48, 2000. doi:10.1038/sj.ijo.0801083. 10702749

[30] Arafat AM, Kaczmarek P, Skrzypski M, Pruszyńska-Oszmalek E, Kołodziejski P, Szczepankiewicz D, Sassek M, Wojciechowicz T, Wiedenmann B, Pfeiffer AF, Nowak KW, Strowski MZ. Glucagon increases circulating fibroblast growth factor 21 independently of endogenous insulin levels: a novel mechanism of glucagon-stimulated lipolysis? Diabetologia 56: 588–597, 2013. doi:10.1007/s00125-012-2803-y. 23262585

[31] Cyphert HA, Alonge KM, Ippagunta SM, Hillgartner FB. Glucagon stimulates hepatic FGF21 secretion through a PKA- and EPAC-dependent posttranscriptional mechanism. PLoS One 9: e94996, 2014. doi:10.1371/journal.pone.0094996. 24733293 PMC3986400

[32] Habegger KM, Stemmer K, Cheng C, Müller TD, Heppner KM, Ottaway N, Holland J, Hembree JL, Smiley D, Gelfanov V, Krishna R, Arafat AM, Konkar A, Belli S, Kapps M, Woods SC, Hofmann SM, D'Alessio D, Pfluger PT, Perez-Tilve D, Seeley RJ, Konishi M, Itoh N, Kharitonenkov A, Spranger J, DiMarchi RD, Tschöp MH. Fibroblast growth factor 21 mediates specific glucagon actions. Diabetes 62: 1453–1463, 2013. doi:10.2337/db12-1116. 23305646 PMC3636653

[33] Alonge KM, Meares GP, Hillgartner FB. Glucagon and insulin cooperatively stimulate fibroblast growth factor 21 gene transcription by increasing the expression of activating transcription factor 4. J Biol Chem 292: 5239–5252, 2017. doi:10.1074/jbc.M116.762922. 28188284 PMC5392671

[34] Welles JE, Dennis MD, Jefferson LS, Kimball SR. Glucagon-dependent suppression of mTORC1 is associated with upregulation of hepatic FGF21 mRNA translation. Am J Physiol Endocrinol Physiol 319: E26–E33, 2020. doi:10.1152/ajpendo.00555.2019. 32421369 PMC7468783

[35] Blomhoff R, Andersen R, Arnesen EK, Christensen JJ, Eneroth H, Erkkola M, Gudanaviciene I, Halldórsson TI, Høyer-Lund A, Lemming EW, Meltzer HM, Pitsi T, Schwab U, Siksna I, Thórsdottir I, Trolle E. Nordic Nutrition Recommendations 2023. Copenhagen, Denmark: Nordic Council of Ministers, 2023.

[36] U.S. Department of Agriculture and U.S. Department of Health and Human Services. Dietary Guidelines for Americans, 2020–2025 (9th ed.). December 2020. Available at DietaryGuidelines.gov.

[37] Laeger T, Albarado DC, Burke SJ, Trosclair L, Hedgepeth JW, Berthoud HR, Gettys TW, Collier JJ, Münzberg H, Morrison CD. Metabolic responses to dietary protein restriction require an increase in FGF21 that is delayed by the absence of GCN2. Cell Rep 16: 707–716, 2016. doi:10.1016/j.celrep.2016.06.044. 27396336 PMC4956501

[38] De Sousa-Coelho AL, Marrero PF, Haro D. Activating transcription factor 4-dependent induction of FGF21 during amino acid deprivation. Biochem J 443: 165–171, 2012. doi:10.1042/BJ20111748. 22233381

[39] Condon KJ, Sabatini DM. Nutrient regulation of mTORC1 at a glance. J Cell Sci 132: jcs222570, 2019. doi:10.1242/jcs.222570. 31722960 PMC6857595

[40] Cornu M, Oppliger W, Albert V, Robitaille AM, Trapani F, Quagliata L, Fuhrer T, Sauer U, Terracciano L, Hall MN. Hepatic mTORC1 controls locomotor activity, body temperature, and lipid metabolism through FGF21. Proc Natl Acad Sci USA 111: 11592–11599, 2014. doi:10.1073/pnas.1412047111. 25082895 PMC4136616

[41] Fisher FM, Kim M, Doridot L, Cunniff JC, Parker TS, Levine DM, Hellerstein MK, Hudgins LC, Maratos-Flier E, Herman MA. A critical role for ChREBP-mediated FGF21 secretion in hepatic fructose metabolism. Mol Metab 6: 14–21, 2017. doi:10.1016/j.molmet.2016.11.008. 28123933 PMC5220398

[42] Iroz A, Montagner A, Benhamed F, Levavasseur F, Polizzi A, Anthony E, Régnier M, Fouché E, Lukowicz C, Cauzac M, Tournier E, Do-Cruzeiro M, Daujat-Chavanieu M, Gerbal-Chalouin S, Fauveau V, Marmier S, Burnol AF, Guilmeau S, Lippi Y, Girard J, Wahli W, Dentin R, Guillou H, Postic C. A specific ChREBP and PPARα cross-talk is required for the glucose-mediated FGF21 response. Cell Rep 21: 403–416, 2017. doi:10.1016/j.celrep.2017.09.065. 29020627 PMC5643524

[43] Sengupta S, Peterson TR, Laplante M, Oh S, Sabatini DM. mTORC1 controls fasting-induced ketogenesis and its modulation by ageing. Nature 468: 1100–1104, 2010. doi:10.1038/nature09584. 21179166

[44] Zhen EY, Jin Z, Ackermann BL, Thomas MK, Gutierrez JA. Circulating FGF21 proteolytic processing mediated by fibroblast activation protein. Biochem J 473: 605–614, 2016. doi:10.1042/BJ20151085. 26635356 PMC4764976

[45] Simpson SJ, Raubenheimer D. Obesity: the protein leverage hypothesis. Obes Rev 6: 133–142, 2005. doi:10.1111/j.1467-789X.2005.00178.x. 15836464

[46] Dhillon J, Craig BA, Leidy HJ, Amankwaah AF, Osei-Boadi Anguah K, Jacobs A, Jones BL, Jones JB, Keeler CL, Keller CE, McCrory MA, Rivera RL, Slebodnik M, Mattes RD, Tucker RM. The effects of increased protein intake on fullness: a meta-analysis and its limitations. J Acad Nutr Diet 116: 968–983, 2016. doi:10.1016/j.jand.2016.01.003. 26947338

[47] Holst JJ, Wewer Albrechtsen NJ, Pedersen J, Knop FK. Glucagon and amino acids are linked in a mutual feedback cycle: the liver-α-cell axis. Diabetes 66: 235–240, 2017. doi:10.2337/db16-0994. 28108603

[48] Ang T, Bruce CR, Kowalski GM. Postprandial aminogenic insulin and glucagon secretion can stimulate glucose flux in humans. Diabetes 68: 939–946, 2019. doi:10.2337/db18-1138. 30833465

[49] Claessens M, Calame W, Siemensma AD, van Baak MA, Saris WH. The effect of different protein hydrolysate/carbohydrate mixtures on postprandial glucagon and insulin responses in healthy subjects. Eur J Clin Nutr 63: 48–56, 2009. doi:10.1038/sj.ejcn.1602896. 17851462

[50] Boirie Y, Dangin M, Gachon P, Vasson MP, Maubois JL, Beaufrère B. Slow and fast dietary proteins differently modulate postprandial protein accretion. Proc Natl Acad Sci USA 94: 14930–14935, 1997. doi:10.1073/pnas.94.26.14930. 9405716 PMC25140

[51] Nilsson M, Stenberg M, Frid AH, Holst JJ, Björck IM. Glycemia and insulinemia in healthy subjects after lactose-equivalent meals of milk and other food proteins: the role of plasma amino acids and incretins. Am J Clin Nutr 80: 1246–1253, 2004. doi:10.1093/ajcn/80.5.1246. 15531672

[52] Fisher FM, Chui PC, Antonellis PJ, Bina HA, Kharitonenkov A, Flier JS, Maratos-Flier E. Obesity is a fibroblast growth factor 21 (FGF21)-resistant state. Diabetes 59: 2781–2789, 2010. doi:10.2337/db10-0193. 20682689 PMC2963536

[53] Solon-Biet SM, Cogger VC, Pulpitel T, Heblinski M, Wahl D, McMahon AC, Warren A, Durrant-Whyte J, Walters KA, Krycer JR, Ponton F, Gokarn R, Wali JA, Ruohonen K, Conigrave AD, James DE, Raubenheimer D, Morrison CD, Le Couteur DG, Simpson SJ. Defining the nutritional and metabolic context of FGF21 using the geometric framework. Cell Metab 24: 555–565, 2016. doi:10.1016/j.cmet.2016.09.001. 27693377

